# Vancomycin, Daptomycin, Antistaphylococcal β-Lactam, and Trimethoprim-Sulfamethoxazole Monotherapy and Combination Therapy in the Management of Methicillin-Resistant *Staphylococcus aureus*: A Network Meta-Analysis

**DOI:** 10.3389/fphar.2022.805966

**Published:** 2022-05-17

**Authors:** Xiaonan Xu, Ni Lu, Pan Song, Mingzhen Zhou, Yuanxiao Li, Zirui Wang, Xin Gao

**Affiliations:** ^1^ Department of Pediatrics, Second Hospital of Lanzhou University, Lanzhou, China; ^2^ The Clinical Medical College of Lanzhou University, Lanzhou, China; ^3^ Department of Urology, Institution of Urology, West China Hospital of Sichuan University, Chengdu, China

**Keywords:** vancomycin, daptomycin, β-lactams, network meta-analysis, MRSA

## Abstract

**Objective:** The aim was to evaluate the efficacy and safety of vancomycin or daptomycin (VAN/DAP), antistaphylococcal β-lactam (ASBL), trimethoprim-sulfamethoxazole (TMP-SMX), and combination therapy of VAN/DAP + ASBL in the management of methicillin-resistant *Staphylococcus* aureus (MRSA).

**Methods:** Databases including PubMed, Cochrane Library, Embase database, and google scholar were searched on 1 September 2021. The randomized control trials (RCTs) and comparable clinical studies of VAN/DAP, VAN/DAP + ASBL, ASBL, and TMP-SMX in the management of MRSA were identified. A network meta-analysis was conducted with STATA 14.0.

**Results:** Seven RCTs and two matched cohorts with 1,048 patients were included in the analysis. The pooled results showed that VAN/DAP + ASBL had a significantly lower rate of persistent bacteremia >3 days than VAN/DAP alone [OR:0.46, 95%CI (0.26, 0.81), *p* < 0.001]. No obvious differences were observed in the outcomes of all-cause mortality, relapsed bacteremia, microbiological treatment failure, embolic or metastatic infection, and total adverse events. However, the ranking results showed that VAN/DAP + ASBL had slightly better efficacy (all-cause mortality, persistent bacteremia >3 days, duration of bacteremia, microbiological treatment failure, and relapsed bacteremia) but slightly higher adverse events than VAN/DAP alone. No obvious differences in the comparisons of VAN/DAP vs. ASBL, and VAN/DAP vs TMP-SMX in the analyzed outcomes. The ranking results revealed that ASBL and TMP-SMX did not have better efficacy or lower adverse events compared with the treatment of VAN/DAP.

**Conclusion:** The efficacy of VAN/DAP + ASBL was slightly but not significantly better than VAN/DAP alone in the management of MRSA.

## Introduction

Antibiotic resistance is a major global health problem, and methicillin-resistant *Staphylococcus aureus* is a serious threat among Gram-positive drug-resistant bacteria. (Vestergaard et al., 2019)According to its original source, MRSA can be divided into hospital-acquired MRSA and community-acquired MRSA. In China, the proportion of MRSA obtained by hospitals has reached 50.4%. (Shang et al., 2016)The proportion of methicillin-resistant *Staphylococcus aureus* in Europe decreased from 26.6% in 2007 to 16.8% in 2015, but it is estimated that the incidence of MRSA infection in the European Union / European Economic area actually increased by 1.28 times over the same period. (Borg and Camilleri, 2021)It has high morbidity and mortality and can cause metastatic or complex infections such as infective endocarditis or septicemia. (Hassoun et al., 2017)It seriously endangers the life and health of patients and causes huge economic losses and heavy burdens to the global medical system.

Currently, the standard treatment for MRSA bacteremia is vancomycin or daptomycin (VAN/DAP) (Liu et al., 2011). However, their efficacy is limited and there are many disadvantages such as poor tissue permeability and slow killing time (Rybak, 2006). Some alternative medicines such as trimethoprim-sulfamethoxazole (TMP-SMX) and ceftaroline have been evaluated in the treatments of MRSA in recent years (Campbell et al., 2012; Holmes et al., 2015). Another strategy for treating MRSA infection is to combine the standard regimen with other drugs. Many *in vitro* data studies have demonstrated that VAN/DAP in combination with antistaphylococcal β-lactam (ASBL) has a synergistic effect on MRSA strains, which can increase the speed of bacterial killing (Holmes et al., 2015). Whether the combination of VAN/DAP with β-lactam has obvious better outcomes than VAN/DAP is still inconclusive.

Network meta-analysis is a new meta-analysis method that uses the evidence of comprehensive randomized trials to evaluate the relative effectiveness of two or more interventions (Cipriani et al., 2013). The purpose of this study is to evaluate the efficacy and safety of VAN/DAP, VAN/DAP + ASBL, ASBL, and TMP-SMX in the management of MRSA.

## Methods

### Search Strategy and Study Selection

A systematic review was performed based on the guidelines for the Preferred Reporting Items for Systematic Reviews and Meta-Analyses (PRISMA) Statement (Page et al., 2021). Four electronic databases including PubMed, Cochrane Library, Web of Science, and Embase database were searched for the clinical studies of VAN/DAP in the management of MRSA from inception to 1 September 2021. MeSH terms and related synonyms including “*staphylococcus aureus*,” “*bacteremia*”, “*Staphylococcus aureus bacteremia*”, “*vancomycin*,” “*vancocine*,” “*vancomicina*,” “*daptomycin*,” “*cubicin*,” and “*LY146032*” were combined and searched. No language limitation was used while searching the databases. All records we retrieved were imported into Endnote X9, and duplicate records were removed by using this software and manually. Two researchers assessed the relevance of each article by reading the title and abstract individually. According to the selection criteria, full-text scanning was conducted to evaluate the eligibility of each related study. Any discrepancies and inconsistencies during the entire evaluation process of the two researchers were resolved by discussion or inquiring with a third researcher.

### Selection Criteria

The trials were included when they met the following criteria: (Vestergaard et al., 2019) the article types were RCTs or clinical studies with comparable basic characteristics; (Shang et al., 2016) patients were diagnosed with MRSA by one or more positive blood cultures for *S. aureus* within two calendar days; (Borg and Camilleri, 2021) the treatment in the control group was using VAN or DAP; treatment in the intervention group was using one of the VAN/DAP + ASBL, ASBL, or TMP-SMX; ASBL was a class of drugs but not specific drugs.

Studies were excluded if they met the following conditions: (Vestergaard et al., 2019) significant difference existed in the basic characteristic of two group of patients; (Shang et al., 2016) patients were associated with other diseases such as the liver and kidney insufficiency; (Borg and Camilleri, 2021) patients were infected with MSSA rather than MRSA; (Hassoun et al., 2017) some other treatments besides those we mentioned above were added during the treatment; (Liu et al., 2011) the study did not provide raw data in the article and we could not obtain it by contacting the corresponding author; (Liu et al., 2011) the outcomes were rare and no other study had similar outcomes; we were unable to conduct a combination of analyses.

### Data Collection and Quality Assessment

For each included study, two independent researchers collected all available data from the article. The information including the first author’s name, year of publication, journal, study design, treatments in experimental and control groups, number of patients in each group, and reported outcomes were collected and collated in Microsoft Excel 2016. The important outcomes which were reported in three or more studies and measured in the same way were pooled and defined as the outcomes of this study. The primary outcomes of this study were all-cause mortality and total adverse events, and the secondary outcomes included duration of bacteremia days, microbiological treatment failure, relapsed bacteremia, and embolic or metastatic infection.

The quality of the included studies was assessed by two independent reviewers based on the tools provided in version 5.1.3 of the Cochrane Handbook. Each trial was evaluated and scored according to the following criteria: random sequence generation, the blindness of participants and result evaluators, incomplete result data reporting, allocation concealment, selective data result reporting, and other deviations. The disagreement between the two reviewers was resolved through discussion or inquiry with the third reviewer.

### Data Synthesis and Analysis

Odds ratio (OR) and 95% confidence interval (95% CI) were used for the statistical analysis of binary variables, and weighted mean difference (WMD) and 95% CI were used for the analysis of continuous variables. The random effect model was adopted in the analysis because inconsistency might exist between different trials and treatments. The comparisons between every two interventions for every outcome were conducted. The ranking analysis which could rank the best to worst interventions for every outcome was introduced to distinguish the best treatment. The consistency analysis was evaluated by the node-splitting analysis and the publication bias was accessed by funnel plots. All analyses were conducted with the software of STATA version 14.0. The packages of “network,” “mvmeta,” and “netfunnel” were used during the analyses . *p* < 0.05 was regarded as a statistical difference.

## Results

### Baseline Characteristics of Included Studies

**FIGURE 1:**
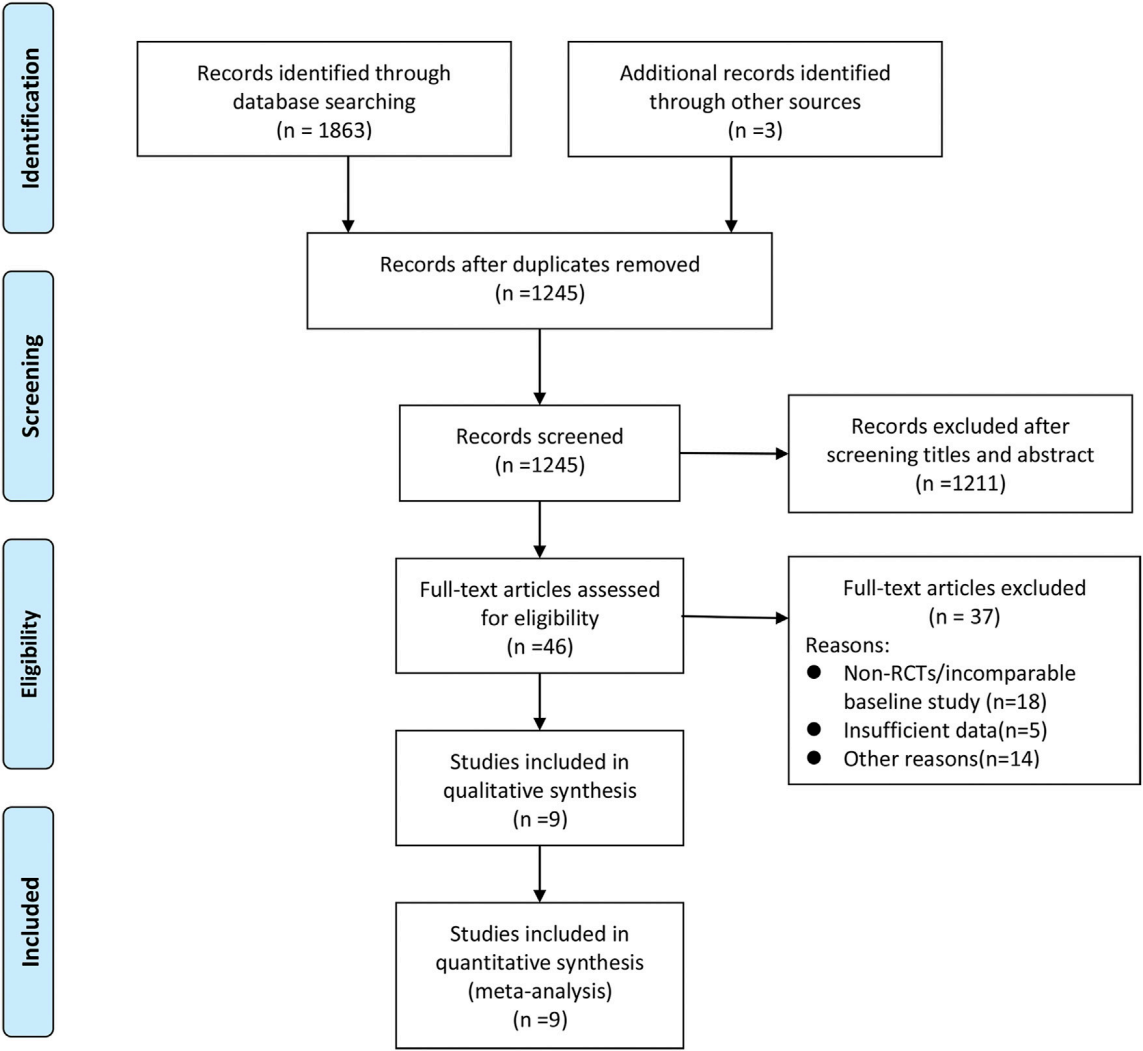
PRISMA flow diagram of the study selection process for network meta-analysis.

A total of 1866 records were retrieved and screened first by reading the titles and abstracts. The remaining 46 articles were evaluated by full-text scanning. Nine studies (Fowler et al., 2006; Dilworth et al., 2014; Paul et al., 2015; Davis et al., 2016; Pericàs et al., 2018; Geriak et al., 2019; Hamed et al., 2020; McCreary et al., 2020; Tong et al., 2020) with 1,048 patients were finally enrolled into our analysis. The whole process of the literature search and screening is shown in Figure 1. The baseline characteristics of the included studies are presented in Table 1.

**TABLE 1:** Characteristics of the included randomized controlled trials in network meta-analysis.

Authors	Year	Journal	Study design	Experimental group	Control group	Samples (E/C)	Main outcomes
Tong et al. (2020)	2020	JAMA	RCT	VAN/DAP + ASBL (flucloxacillin, cloxacillin, or cefazolin)	VAN/DAP	174/178	All-cause mortality; persistent bacteremia; relapsed bacteremia; microbiological treatment failure; adverse events
Geriak et al. (Geriak et al. (2019)	2019	Clinical Infectious Diseases	RCT	VAN/DAP + ASBL (ceftaroline)	VAN/DAP	17/23	All-cause mortality; persistent bacteremia; median duration of bacteremia days; adverse events
Davis et al. (2016)	2015	Antimicrob Agents Chemother	RCT	VAN/DAP + ASBL (flucloxacillin)	VAN/DAP	31/29	All-cause mortality; median duration of bacteremia days; relapsed bacteremia; embolic or metastatic infection; adverse events
McCreary et al. (2020)	2019	Open Forum Infectious Diseases	MatchedCohort	VAN/DAP + ASBL (ceftaroline)	VAN/DAP	58/113	All-cause mortality; median duration of bacteremia days; microbiological treatment failure;
Dilworth et al. (2014)	2014	Antimicrob Agents Chemotherapy	MatchedCohort	VAN/DAP + ASBL (ceftaroline)	VAN/DAP	50/30	All-cause mortality; microbiological eradication
Hamed et al. (2020)	2020	Future Microbiol	RCT	VAN/DAP	ASBL (ceftobiprole)	195/195	All-cause mortality; microbiological eradication; relapsed bacteremia; embolic or metastatic infection; adverse events
Pericàs et al. (2018)	2018	Clinical Microbiology and Infection	RCT	VAN/DAP	ASBL (imipenem)	7/8	All-cause mortality; persistent bacteremia; adverse events
Fowler et al. (2006)	2006	New England Journal of Medicine	RCT	VAN/DAP	ASBL (nafcillin, oxacillin, or flucloxacillin)	120/116	The median duration of bacteremia days; adverse events
Paul et al. (2015)	2015	BMJ	RCT	TMP-SMX	VAN/DAP	53/51	All-cause mortality; persistent bacteremia; microbiological treatment failure; adverse events

E/C, Experimental group versus control group; JAMA, Journal of the American medical association; BMJ, British medical journal; RCT, randomized controlled trial; VAN/DAP, vancomycin or daptomycin; ASBL, antistaphylococcal β-lactam; TMP-SMX, Trimethoprim-sulfamethoxazole.

**FIGURE 2:**
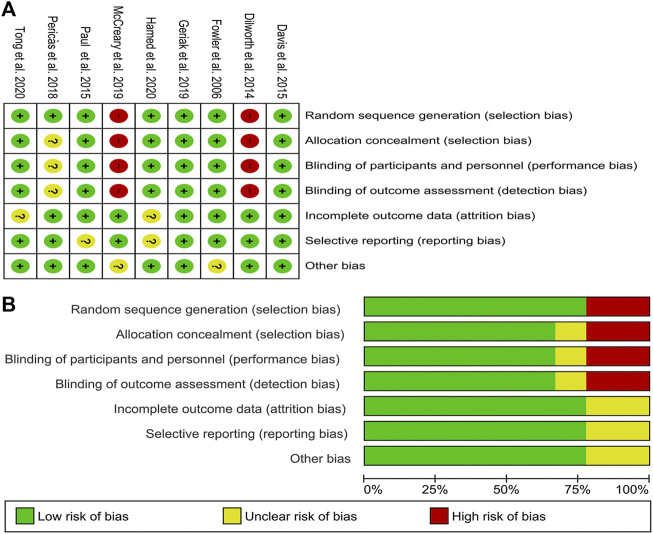
Risk of bias graph and summary of the included studies **(A)** reviewers’ judgments about each risk of bias item for eligible studies and **(B)** the judgments about each risk of bias item presented as percentages across all eligible studies.

The included studies were seven RCTs and two matched cohorts with comparable basic characteristics. Most of the RCTs were randomized groups and described adequate randomization in detail. The detailed information about allocation sequence concealment was well described in most of the included RCTs. Attrition bias and reporting bias were well-performed. The risk-of-bias assessment is summarized in Figure 2.

### Network Meta-Analysis of All-Cause Mortality

**FIGURE 3:**
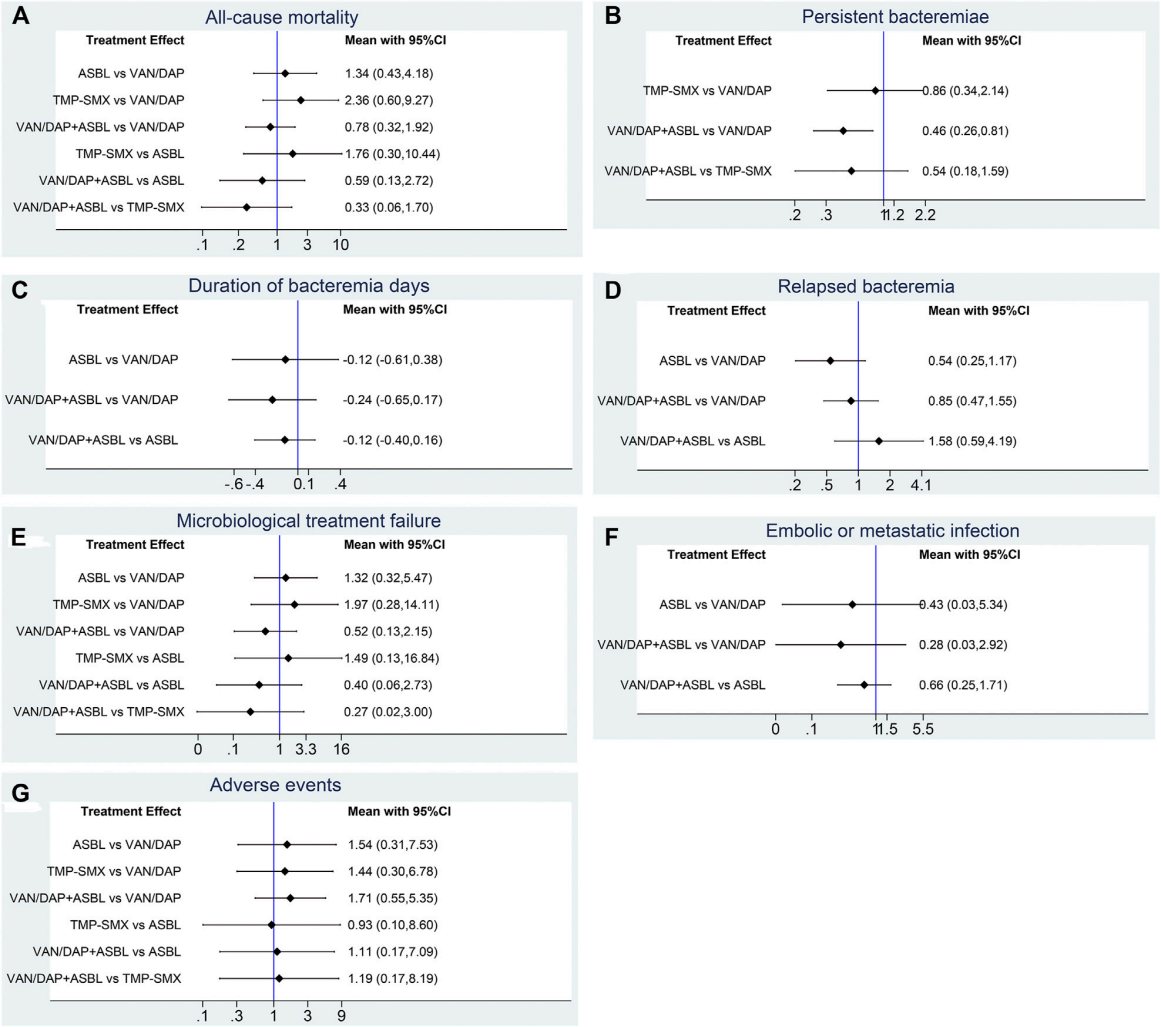
Forest plots for the outcomes. **(A)** All-cause mortality; **(B)** Persistent bacteremia; **(C)** Duration of bacteremia days; **(D)** Relapsed bacteremia ; **(E)** Microbiological treatment failure; **(F)** Embolic or metastatic infection; **(G)** Adverse events. VAN/DAP, Vancomycin or daptomycin; ASBL, antistaphylococcal β-lactam; TMP-SMX, trimethoprim-sulfamethoxazole.

**FIGURE 4:**
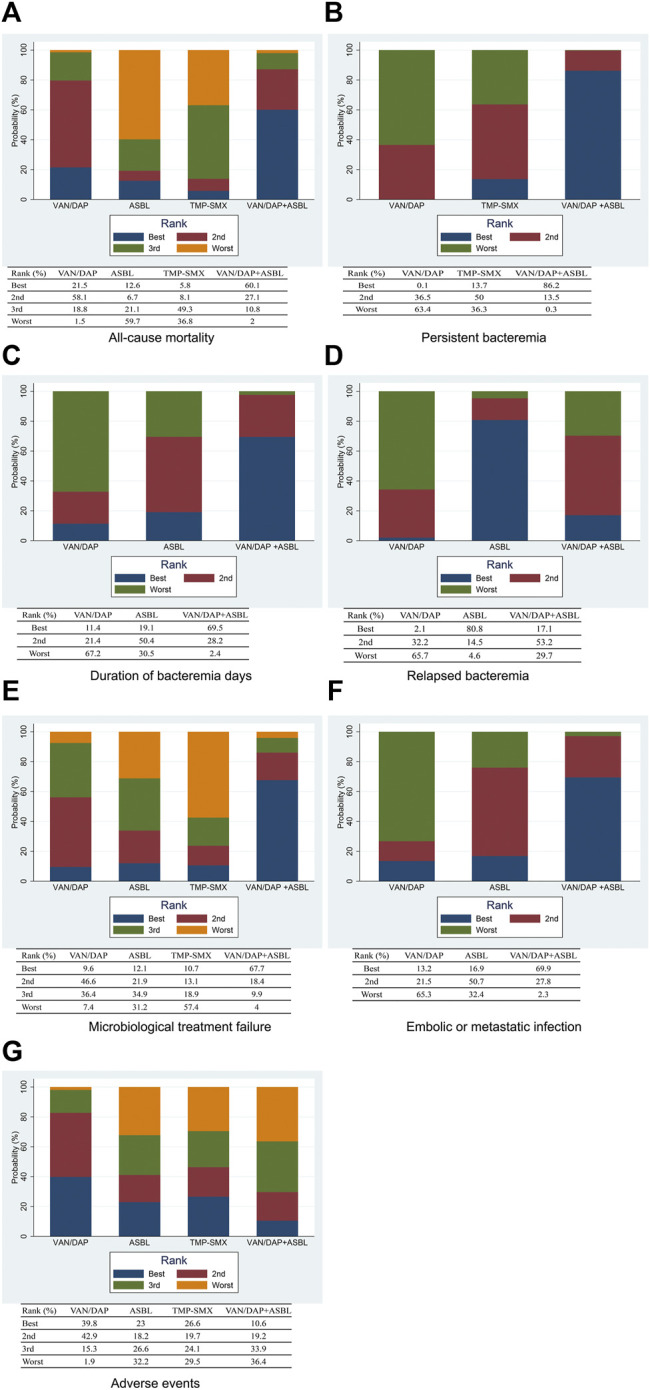
The ranking plots for the efficacy outcomes **(A)** All-cause mortality; **(B)** persistent bacteremia; **(C)** Duration of bacteremia days; **(D)** Relapsed bacteremia; **(E)** Microbiological treatment failure; **(F)** Embolic or metastatic infection; **(G)** Adverse events. VAN/DAP, Vancomycin or daptomycin; ASBL, antistaphylococcal β-lactam; TMP-SMX, trimethoprim-sulfamethoxazole.

Seven studies with 951 patients described the outcome of all-cause mortality. No significant differences were detected among the four treatments. With VAN/DAP as the reference, the ORs and 95%CIs of VAN/DAP + ASBL, ASBL, and TMP-SMX were 0.78(0.32, 1.92), 1.34 (0.43, 4.18), and 2.36 (0.60, 9.27), individually. These results are presented in Figure 3A. Ranking results for these interventions showed that VAN/DAP + ASBL was the best and was slightly better than other treatments. ASBL was the worst for this outcome (Figure 4A).

### Network Meta-Analysis of Persistent Bacteremia

Four studies with 489 patients reported the number of patients with persistent bacteremia for more than 3 days. Results showed that VAN/DAP + ASBL had a significantly lower rate of persistent bacteremia longer than 3 days than VAN/DAP [OR = 0.51, 95%CI (0.29, 0.91), *p* < 0.001]. With VAN/DAP as the reference, the OR and 95% CI of ASBL and TMP-SMX were 1.09 (0.20, 5.94) and 0.86 (0.34, 2.14). All results are presented in Figure 3B. The ranking results revealed that VAN/DAP + ASBL ranked 1st and was the best treatment in this outcome (Figure 4B).

Six studies described the duration of bacteremia days. There were no significant differences among VAN/DAP, VAN/DAP + ASBL, and TMP-SMX. With VAN/DAP as the reference, the SMD and 95%CI of VAN/DAP + ASBL and TMP-SMX were 0.46 (0.26, 0.81) and 0.86(0.34,2.14). The ranking results revealed that VAN/DAP + ASBL had the shortest duration of bacteremia days and was slightly better than the other treatments. All these results are shown in Figure 4C.

### Network Meta-Analysis of Relapsed Bacteremia

Six trials with 879 patients were analyzed. Results suggested that there were no significant differences among VAN/DAP, VAN/DAP + ASBL, and ASBL. With VAN/DAP as the reference, the OR and 95%CI of VAN/DAP + ASBL and ASBL were 0.85(0.47, 1.55) and 0.54(0.25, 1.17). The ranking results showed that ASBL was the best, and VAN/DAP + ASBL ranked 2nd. All these results are shown in Figure 4D.

### Network Meta-Analysis of Microbiological Treatment Failure

Five trials with 604 patients were pooled in this analysis. No significant differences were found among VAN/DAP, VAN/DAP + ASBL, ASBLA, and TMP-SMX. With VAN/DAP as the reference, the OR and 95%CI of VAN/DAP + ASBL, ASBL, and TMP-SMX were 0.52 (0.13, 2.15), 1.32(0.32, 5.47), and 1.97 (0.28, 14.11), respectively. These results are presented in Figure 3E. The ranking results revealed that VAN/DAP + ASBL was the best. VAN/DAP ranked 2nd (Figure 4E).

### Network Meta-Analysis of Embolic or Metastatic Infection

Three studies reported embolic or metastatic infection, recruiting 158 patients. The pooled estimates showed that the differences among VAN/DAP + ASBL, VAN/DAP, and ASBL were not significant. With VAN/DAP as the reference, the OR and 95%CI of VAN/DAP + ASBL and ASBL were 0.28(0.03, 2.92) and 0.43(0.03, 5.34). The ranking results showed that VAN/DAP + ASBL was the best. These results are presented in Figure 4F.

### Network Meta-Analysis of Adverse Events

Seven trials described the total number of adverse events and were combined. Results showed that there were no significant differences in the incidence of any adverse events among the four treatments. With VAN/DAP as the reference, the OR and 95%CI of VAN/DAP + ASBL, ASBL, and TMP-SMX were 1.71 (0.55, 5.35), 1.54 (0.31, 7.53), and 1.44(0.30, 6.78), respectively. These results are shown in Figure 3G. The ranking results revealed that VAN/DAP ranked 1st, TMP-SMX ranked 2nd, VAN/DAP + ASBL ranked 3^rd^, and ASBL was the worst (Figure 4G).

### Consistency Analysis

The node-splitting analysis was adopted to evaluate inconsistency by comparing the differences between direct and indirect evidence. The publication bias was evaluated by funnel plots. No obvious inconsistency and publication bias were detected in all outcomes.

## Discussion

From the comparisons of VAN/DAP and the combination therapy of VAN/DAP + ASBL, we found that (Vestergaard et al., 2019) VAN/DAP + ASBL significantly reduced the number of patients with persistent bacteremia >3 days. (Shang et al., 2016) No obvious difference was observed between VAN/DAP + ASBL and VAN/DAP in the outcomes of all-cause mortality, relapsed bacteremia, microbiological treatment failure, embolic or metastatic infection, and adverse events. (Borg and Camilleri, 2021) The ranking results revealed that VAN/DAP + ASBL was slightly better than VAN/DAP alone in all-cause mortality, persistent bacteremia >3 days, duration of bacteremia, microbiological treatment failure, and relapsed bacteremia.

Some previous studies reported similar outcomes. Atalla and Mylonakis (2020) pointed out that the blood culture bacteria clearance rate of the combined group (VAN/DAP + ASBL) was faster than that of the standard group (VAN/DAP). Hagihara et al. (2012) reported that the combination of vancomycin and ASBL in simulated human exposure increased the killing rate of these MRSA isolates and resulted in a greater overall antibacterial effect. Holubar suggested that the combination of vancomycin or daptomycin with β-lactam antibiotics was associated with a shorter duration of bacteremia, but there was no significant clinical benefit. (Holubar et al., 2020) However, Wang pointed out that the combination therapy has certain clinical benefits, but it may be related to the use of a certain type of beta-lactam drugs in the experimental group, such as ceftaroline, and perhaps ceftaroline itself has anti-MRSA effects. (Wang et al., 2020) Some scholars believe that the clinical effect of combined therapy is related to the length of treatment time and the concentration of IL-10 in the blood. (Geriak et al., 2019; Johnson et al., 2021) MRSA is naturally resistant to β-lactam drugs, resistance is usually conferred by the acquisition of a nonnative gene encoding a penicillin-binding protein (PBP2a), with a significantly lower affinity for β-lactams. This resistance allows cell-wall biosynthesis, the target of β-lactams, to continue even in the presence of typically inhibitory concentrations of antibiotics (Peacock and Paterson, 2015), and the significant changes in the cell-wall composition required for vancomycin resistance make these strains resistant to β-lactams antibiotics are more sensitive (Domenech et al., 2005). *In vitro* studies have demonstrated synergistic interactions between vancomycin and a wide variety of β-lactams against *S. aureus* (Fox et al., 2006; Ortwine et al., 2013). The proposed mechanism for vancomycin and β-lactam synergy is related to cell-wall thinning via the addition of a β-lactam, which increases vancomycin binding to target sites during cell wall synthesis (Sarkar et al., 2017). A study found that adding ASBL to VAN/DAP therapy for at least 24 h within 72 h could increase the odds of clinical success (Alosaimy et al., 2020).

As for the results of TMP-SMX and ASBL in the treatment of MRSA. Even though no significant differences were detected with VAN/DAP, the ranking results showed that ASBL and TMP-SMX were less efficient than VAN/DAP in most of the outcomes. Some previous studies had reported similar results. Tissot-Dupont et al. (2019) reported that TMP-SMX did not achieve noninferiority compared with vancomycin among patients with invasive MRSA infections. There were also some different conclusions. Eliakim-Raz et al. (2017) pointed out that TMP-SMX had an efficacy profile superior to that of vancomycin in the treatment of MRSA pneumonia, with lower rates of overall mortality and the same safety profile. As for ASBL, it is often adopted in the treatment of MSSA. It was reported that Cefazolin and Ertapenem could rapidly clear persistent MSSA (Ulloa et al., 2020). Recently, it was also explored in the management of MRSA. Ceftaroline and ceftobiprole are the first β-lactams with antiMRSA activity (Lee et al., 2018). Many *in vitro* studies (Espedido et al., 2015; Sader et al., 2015) had demonstrated the potent *in vitro* activity of Ceftaroline against MRSA isolates causing bacteremia. In a matched clinical cohort study, Arshad et al. (2017) compared Ceftaroline fosamil with VAN and DAP for MRSA bacteremia. They found that Ceftaroline fosamil, especially as salvage therapy, had a longer duration of bacteremia but comparable clinical outcomes with VAN/DAP for MRSA bacteremia patients. These results were somewhat similar to our results.

Our meta-analysis had certain limitations. First, although the quality of the studies we included was relatively high, the number of studies included in our analysis was insufficient, which may affect the accuracy of the results. Second, some of the outcome indicators included in the study were not perfect which made it impossible to combine and analyze these results. It might cause incomplete analysis results or omission of some results. Third, the follow-up time of included studies was quite different. Some of the included studies only followed-up for a short period which might influence the pooled results. Therefore, more high-quality studies are needed in the future to evacuate the results of our study.

## Conclusion

VAN/DAP + ASBL had slightly but not significantly better efficacy than VAN/DAP alone in the treatment of MRSA. ASBL and TMP-SMX didn’t have better efficacy or lower adverse events than VAN/DAP.

## Data Availability

The original contributions presented in the study are included in the article/Supplementary Material; further inquiries can be directed to the corresponding author.
